# High‐Pressure Synthesis of Ultra‐Incompressible Beryllium Tungsten Nitride Pernitride BeW_10_N_14_(N_2_)

**DOI:** 10.1002/anie.202505778

**Published:** 2025-04-10

**Authors:** Georg Krach, Lukas Brüning, Sebastian Ambach, Elena Bykova, Nico Giordano, Björn Winkler, Maxim Bykov, Wolfgang Schnick

**Affiliations:** ^1^ Department of Chemistry University of Munich (LMU) Butenandtstraße 5–13 81377 Munich Germany; ^2^ Institute of Inorganic and Analytical Chemistry Goethe University Frankfurt Max‐von‐Laue‐Straße 7 60438 Frankfurt am Main Germany; ^3^ Institute of Geosciences Goethe‐University Frankfurt Altenhoeferallee 1 60438 Frankfurt am Main Germany; ^4^ Deutsches Elektronen‐Synchrotron DESY Notkestr. 85 22607 Hamburg Germany

**Keywords:** Beryllium, Diamond anvil cell, High‐pressure synthesis, Pernitride, Ultra‐incompressible

## Abstract

For the activation of nitrogen and its reduction to ammonia, transition metals are crucial in biological as well as industrial processes. So far, only a few binary transition metal compounds with nitrogen dimer anions are known, whereas a ternary compound has remained undiscovered as yet. Here, we report on the synthesis and properties of the first ternary transition metal compound, namely, BeW_10_N_14_(N_2_), which exhibits dinitrogen anions. It was synthesized in a high‐temperature high‐pressure approach from W_2_Be_4_N_5_. The crystal structure, elucidated with synchrotron radiation, unites WN_7_ capped trigonal prisms with intriguing BeN_6_ octahedra and (N_2_)‐anions. Elastic and electronic properties of the title compound were corroborated by DFT calculations, revealing simultaneous ultra‐incompressible and metallic behavior. The synthesis and investigation of the first ternary transition metal nitride with dinitrogen units opens the door to a new field of research on nitride and pernitride chemistry.

## Graphical abstract

The first ternary transition metal pernitride was synthesized in a high‐temperature high‐pressure approach starting from W_2_Be_4_N_5_. The structure features not only the rare structural motif of BeN_6_ octahedra but also dinitrogen anions. DFT calculations reveal ultra‐incompressible and metallic behavior of the new compound.

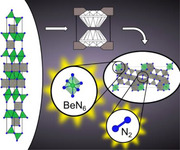

Transition metal catalysts are essential for the activation of nitrogen and its reduction to ammonia. Examples include the well‐known Haber–Bosch process, which uses an iron‐based catalyst, and the enzyme nitrogenase, which contains a Fe–Mo cofactor.^[^ ^1^, ^2^, ^3^, ^4^ ^]^ In the Yandulov–Schrock cycle, a Mo‐based catalyst enables the reduction of the N_2_ molecule in the only artificial process at ambient conditions.^[^ ^5^, ^6^, ^7^ ^]^ The reduction can be followed via several stages represented by the oxidation states of the nitrogen dimer of 0, ‐II or ‐IV. The corresponding molecules/anions would be dinitrogen, diazenide, or hydrazide anions. The latter is isoelectronic to the peroxide ion O_2_^2−^ and hence can be named analogously as pernitride. Many inorganic compounds are known to stabilize these intermediate dinitrogen units, for example, *AE*N_2_ (*AE* = Ca, Sr, Ba)^[^ ^8^, ^9^ ^]^ for the diazenide (N_2_)^2−^ ion or OsN_2_, IrN_2_, PtN_2_, or TiN_2_ for the pernitride anion (N_2_)^4−^.^[^ ^10^, ^11^, ^12^ ^]^ However, recent discoveries show that formal charges of −1 or −3 are also possible for the N_2_ unit as found in LiN_2_ or FeN_2_, respectively.^[^ ^13^, ^14^, ^15^ ^]^ Furthermore, nitrogen dimers with noninteger charges were found in *M*_3_(N_2_)_4_ (*M* = Na, Ca, Sr, Ba).^[^ ^16^, ^17^ ^]^ The “inert” element nitrogen becomes highly reactive under suitable conditions, creating unprecedented structures and polyhedra that have not been previously observed in nitrides;^[^ ^18^ ^]^ for example, BeN_6_ octahedra in the polynitride BeN_4_, synthesized at 84 GPa.^[^ ^19^ ^]^ Although tremendous efforts in the research of binary nitrides, diazenides, pernitrides, and polynitrides have expanded our knowledge in this field, we hardly know anything about ternary compounds with (N_2_)*^x^* ^−^ anions. The only exception is Li_2_Ca_3_(N_2_)_3_,^[^ ^20^ ^]^ which, to the best of our knowledge, is the only ternary compound with dinitrogen units known until now.

Recently, we have reported the synthesis of the first transition metal nitridoberyllates W_2_Be_4_N_5_ and W_4_Be_8_N_9_.^[^ ^21^ ^]^ We raised the question whether the formation of BeN_6_ octahedra is possible in a high‐pressure modification of W_2_Be_4_N_5_ according to the pressure homologue rule in analogy to the structurally related MgWN_2_.^[^ ^22^, ^23^ ^]^ Driven by this question, we have probed the high‐pressure behavior of W_2_Be_4_N_5_. Upon heating of W_2_Be_4_N_5_ at elevated pressure, we discovered BeW_10_N_14_(N_2_), which not only exhibits BeN_6_ octahedra but is also the first ternary transition metal compound to contain dinitrogen anions. The title compound may be seen as the first ternary nitride pernitride as this combination of nitride and dinitrogen anions is so far only known in binary compounds, e.g., in SrN (Sr_8_N_4_(N_2_)_2_), Re(N_2_)N_2_, or Mo_3_(N_2_)N_3_.^[^ ^24^, ^25^, ^26^ ^]^ Li_2_Ca_3_(N_2_)_3_ exhibits solely dinitrogen anions and no nitride ions.^[^ ^20^ ^]^

The starting material, W_2_Be_4_N_5_, was synthesized according to Equation 1 in a large volume press at 8 GPa and 1400 °C.

(1) $$4Be_{3}N_{2}+6W+3\mathrm{Na}N_{3}\to 3W_{2}Be_{4}N_{5}+3\mathrm{Na}+N_{2}$$

In order to confirm the structural model of W_2_Be_4_N_5_ and to identify a suitable crystal for the diamond anvil cell (DAC) experiment, single crystal X‐ray diffraction (sc‐XRD) data were collected at ambient pressure. The integration of diffraction data collected from a strongly scattering domain yielded an appropriate data set, from which the structure of W_2_Be_4_N_5_ was determined (*a* = 2.8702(2), *c* = 37.1324(18) Å; $R\bar{3}m$(no. 166), *R*_1_ = 0.0254).^[^ ^27^ ^]^ The structure obtained here validates the model that we have reported before and clarifies structural details (Figure S1).^[^ ^21^ ^]^ More information on the synthesis and the refined crystal structure of W_2_Be_4_N_5_ can be found in the Supporting Information.

To investigate the elastic properties of W_2_Be_4_N_5_, a DAC was loaded with a single crystal and Ne as pressure transmitting medium. The sample was compressed at ambient temperature in seven steps up to a pressure of 41.0(1) GPa. At each step, sc‐XRD data was collected to elucidate the pressure‐dependence of the lattice parameters and unit cell volume (Figures 1 and S2). The unit cell volume decreases by approximately 11% over the examined pressure range. The pressure–volume data were fitted with a second‐order Birch–Murnaghan equation of state revealing an isothermal bulk modulus *K*_0_ = 273.4(2) GPa with a fixed value for *K*’ = 4.00 (Figure 1).^[^ ^28^ ^]^ The lattice parameters *a* and *c* show a similar compressibility up to approximately 27 GPa (Figure S2). Beyond this pressure point, the *c* axis is more compressible. The evaluation of the sc‐XRD data revealed no pressure‐induced phase transition on compression at ambient temperature. More information on the elastic properties and the DAC setup can be found in the Supporting Information.

**Figure 1:**
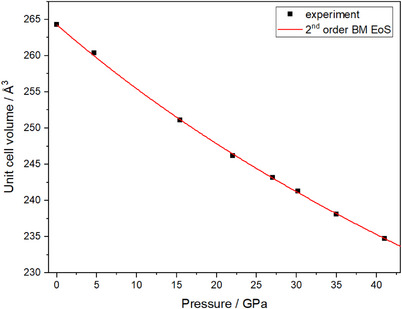
Pressure–volume data from pressure‐dependent single crystal refinements of W_2_Be_4_N_5_ were fitted with a 2nd order Birch–Murnaghan equation of state.

To induce a phase transition or reaction, the single crystal was laser heated from both sides at 41.0(1) GPa (NIR fiber laser, *λ* = 1064 nm) for approximately 30 s. The temperature was likely above 3000 K based on previous observations, but was not precisely determined. The pressure was determined after heating to 42.1(1) GPa. The subsequent XRD scan showed a clearly altered diffraction pattern and the formation of different crystalline phases. One of the new phases was the high‐pressure modification of the binary nitride W_2_N_3_ (*a* = 7.712(7), *b* = 2.7953(5), *c* = 8.059(2) Å; *Pnma* (no. 62)), which we have already reported on elsewhere.^[^ ^29^ ^]^ From the analysis of another dataset, we determined the structure of the first ternary transition metal pernitride BeW_10_N_14_(N_2_). Its structure was solved and refined in space group *C*2*/m* (no. 12) with *a* = 19.803(5), *b* = 2.7867(6), *c* = 8.2467(15) Å, *β* = 99.605(19)°, and *Z* = 2 at 42.1(1) GPa.^[^ ^27^ ^]^

The refined crystal structure and coordination polyhedra are illustrated in Figure 2. The structure consists of WN_7_ single‐capped trigonal prims, regular WN_6_ trigonal prisms, BeN_6_ octahedra, and (N_2_)‐units. The W–N polyhedra are interconnected by vertex‐, edge‐, and surface‐sharing. This structural motif is known from other tungsten nitrides like W_2_N_3_ or W_3_N_5_.^[^ ^29^ ^]^ A special feature is the connection of several WN_7_ capped trigonal prims via the (N_2_) unit, so far only known from W_3_N_5_. The voids in the basic structure formed from tungsten and nitrogen are filled with Be atoms forming edge‐sharing BeN_6_ octahedra that are stacked along the *b* axis. This structural motif has not yet been observed in a nitride compound. The only other phase with BeN_6_ octahedra is BeN_4_, whereas in its structure, the BeN_6_ octahedra are connected via pernitride units.^[^ ^19^ ^]^

**Figure 2:**
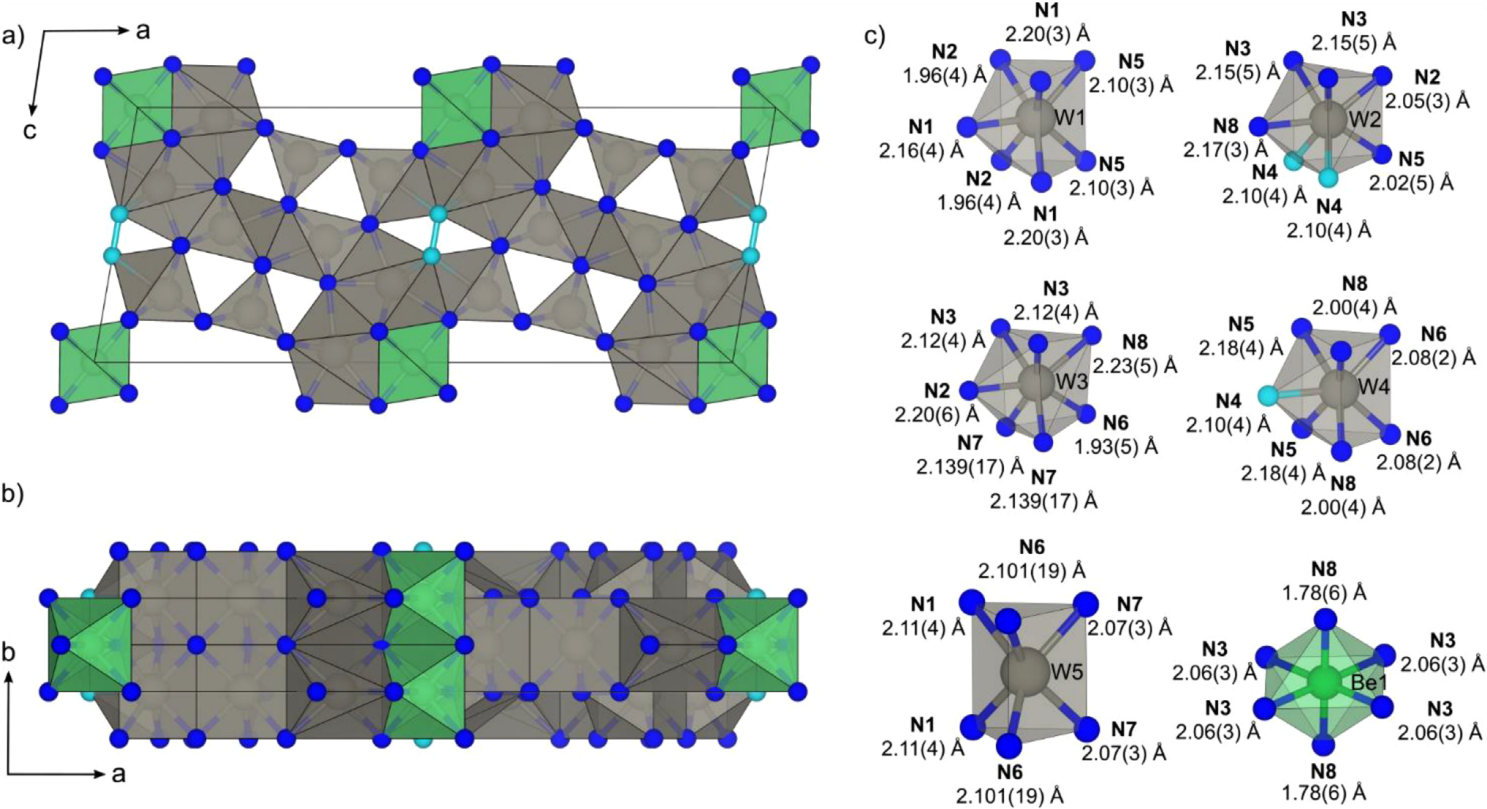
a) and b) Crystal structure and c) coordination polyhedra of cations of BeW_10_N_14_(N_2_) at 16.5(1) GPa. The structure consists of WN_7_ single capped trigonal prisms, regular WN_6_ trigonal prims, and BeN_6_ octahedra. Viewed along the *c* axis, the stacking of the octahedra along *b* via edge sharing becomes apparent (b). Be: green, W: gray, N: blue. The (N_2_)‐dumbbell is cyan colored.

It is remarkable that Be in sixfold coordination can be obtained at the relatively low pressure point of 42.1(1) GPa in a nitride. In comparison, BeN_4_ was synthesized at 84 GPa and undergoes a phase transition when pressure is reduced. The formation of BeO_6_ octahedra is known from a high‐pressure polymorph of the mineral hurlbutite, CaBe_2_(PO_4_)_2_, when compressed above 90 GPa.^[^ ^30^ ^]^ However, Spahr et al. demonstrated that in calcite‐type BeCO_3_, Be in sixfold coordination can be formed already at a pressure of approx. 30 GPa.^[^ ^31^ ^]^ Moreover, Pointner et al. synthesized P_1–_ *_x_* Ta_8+_ *_x_* N_13_ (*x* = 0.1–0.15), which exhibits the common high‐pressure motif of PN_6_ octahedra, previously synthesized at approx. 40 GPa (spinel‐type BeP_2_N_4_ or β‐BP_3_N_6_) at only 8 GPa in a large volume press.^[^ ^32^ ^]^ Considering these recent discoveries, the presence of BeN_6_ octahedra at a pressure of approx. 40 GPa seems plausible.

The distances in the W–N polyhedra (1.96(4)–2.23(5) Å) are consistent with those in W_2_N_3_ (2.02(3)–2.115(19) Å) and W_3_N_5_ (1.954(11)–2.106(5) Å).^[^ ^29^ ^]^ The interatomic distances for BeN_6_ octahedra (1.78(6)–2.06(3) Å) can only be compared to those of BeN_4_ measured at 84 GPa (1.667(4)–1.827(3) Å) and therefore only allow to state that these distances are similar.^[^ ^19^ ^]^ The (N_2_) unit has a bond distance of 1.35(12) Å, in reasonable agreement with distances in FeN_2_ (1.307(10))^[^ ^14^, ^15^ ^]^ or RhN_2_ (1.30(5)).^[^ ^33^, ^34^ ^]^ As no framework with a negative charge built up from BeN_4_ tetrahedra is present, and with respect to the formula BeW_10_N_14_(N_2_), the new compound should be classified as a beryllium tungsten nitride pernitride rather than a tungsten nitridoberyllate, as is appropriate for W_2_Be_4_N_5_.^[^ ^35^ ^]^ More detailed information on the crystal structure of BeW_10_N_14_(N_2_) at different pressure points, reconstructed *hkl* planes, and a Le Bail fit can be found in the Supporting Information.

The weak atomic form factor of Be adjacent to the strongly scattering W and the low completeness of the data set, which is due to the measurement of a monoclinic system in a DAC, raises the question of whether the position of Be atoms can be determined accurately.^[^ ^36^, ^37^ ^]^ However, the refinement of alternative structural models gave unsatisfactory results and were abandoned, but from the experimental data we cannot rule out disorder within the channels. In order to independently confirm the structural model derived from experiment, DFT‐based model calculations were carried out. The full geometry optimizations yielded a relaxed structure in very good agreement with the structural model derived from experiment (see Tables S5–S11 and S13–S17).

Based on this agreement, we computed the enthalpy change due to the incorporation of Be into a theoretical W_10_N_16_, according to Equation 2.

(2) $$\mathrm{Be}+W_{10}N_{16}\to \mathrm{Be}W_{10}N_{16}$$

The incorporation of beryllium, according to Equation 2, is highly preferred with *ΔH* =  −5.348 eV per unit cell, i.e., 256 kJ mol^−1^. Additionally, we have explored if small atoms such as Ne, which was employed as a pressure‐transmitting medium, would be incorporated into the channels. However, the analysis of the sc‐XRD data provided no indication of such an incorporation, and the corresponding DFT calculations implied that, in the athermal limit, such an incorporation would be energetically unfavorable.

Furthermore, the DFT calculations also strengthened the arguments for the presence of a (N_2_) dimer. The computed N–N distance at 40 GPa in the dimer is *d*(N–N) = 1.388 A, in good agreement with the experimental value (1.35(12) Å). The N–N bond has a Mulliken population of 0.65 e^−^ A^3^, i.e., this is a predominantly covalent bond.

In order to answer the question if BeW_10_N_14_(N_2_) is stable at ambient pressure, the sample was decompressed. Although the crystal quality of BeW_10_N_14_(N_2_) degrades with decreasing pressure, we could still observe the phase at ambient pressure. Unfortunately, sc‐XRD data could only be collected at two pressure points during decompression. Hence, the decompression data was complemented with data from the DFT calculations. Figure 3 shows the experimental and theoretical data. The analysis of the DFT compression data gave a bulk modulus of *K*_0_ = 338.5(4) GPa with a pressure derivative of *K*’ = 4.61(3). The experimentally determined unit cell volumes are systematically smaller than those obtained from the DFT calculations. This is due to the underbonding typically encountered in DFT‐GGA‐PBE calculations such as those that have been carried out here. Bulk moduli of DFT‐GGA‐PBE are typically lower than the corresponding experimental values.^[^ ^38^, ^39^ ^]^ The evaluation of the elastic stiffness tensor from stress–strain DFT calculations with the program ELATE revealed an isothermal bulk modulus *K*_0_ = 337 GPa, in very good agreement with the value obtained from the equation of state. Accordingly, BeW_10_N_14_(N_2_) can be described as an ultra‐incompressible material as it has a bulk modulus >300 GPa.^[^ ^40^, ^41^ ^]^ However, high incompressibility does not necessarily correlate with high hardness, which is reflected by the value of the Vickers hardness *H*_v_ of approximately 21 GPa derived from the elastic stiffness tensor.^[^ ^42^ ^]^ The examination of the electronic properties of BeW_10_N_14_(N_2_) using DFT calculations revealed metallic behavior (Figure S7), in line with the results of the only other ternary pernitride Li_2_Ca_3_(N_2_)_3_. More information on DFT calculations and elastic and electronic properties can be found in the Supporting Information.

**Figure 3:**
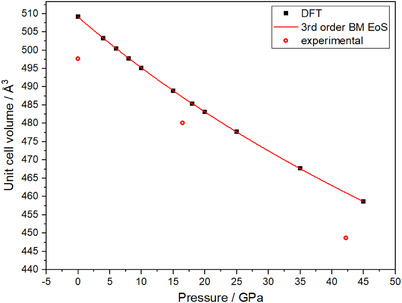
Pressure–volume data from DFT calculations for BeW_10_N_14_(N_2_) fitted with a 3rd order Birch–Murnaghan equation of state. Error bars associated with the experimental data are smaller than the symbol size.

In summary, we have synthesized the first ternary transition metal pernitride BeW_10_N_14_(N_2_) at 42 GPa, which also exhibits BeN_6_ octahedra at far lower synthesis conditions than before (84 GPa for BeN_4_).^[^ ^19^ ^]^ The crystal structure was elucidated in a combined approach from sc‐XRD synchrotron data and DFT calculations. The structure exhibits anions of (N_2_) dimer units that were not observed in a ternary transition metal compound, so far. The examination of combined experimental and theoretical pressure–volume data revealed ultra‐incompressible behavior with a bulk modulus *K*_0_ = 337 GPa. Additionally, the compound is quenchable to ambient pressure. The calculation of the electronic structure reveals metallic properties.

The synthesis of BeW_10_N_14_(N_2_) lays the foundation for the synthesis of further ternary transition metal pernitrides. Due to the structural relationship of Be and Fe in high‐pressure synthesized compounds like BeN_4_ and FeN_4_, the possibility of the synthesis of a ternary iron–tungsten pernitride seems likely.^[^ ^14^, ^19^ ^]^ This model compound could shed light on the nitrogen fixation process and its intermediate steps occurring in the Fe–Mo cofactor in the natural enzyme nitrogenase.^[^ ^3^, ^4^ ^]^

## Supporting Information

The authors have cited additional references within the Supporting Information.^[^ ^43^, ^44^, ^45^, ^46^, ^47^, ^48^, ^49^, ^50^, ^51^, ^52^, ^53^, ^54^, ^55^, ^56^, ^57^, ^58^, ^59^, ^60^, ^61^ ^]^

## Conflict of Interests

The authors declare no conflict of interest.

## Supporting information



Supporting Information

Supporting Information S1

Supporting Information S2

## Data Availability

The data that support the findings of this study are available in the Supporting Information of this article.

## References

[1] J. Kim , D. C. Rees , Biochemistry 1994, 33, 389–397. 8286368 10.1021/bi00168a001

[2] J. Humphreys , R. Lan , S. Tao , Adv. Energ. Sust. Res. 2021, 2, 2000043.

[3] J. B. Howard , D. C. Rees , Chem. Rev. 1996, 96, 2965–2982. 11848848 10.1021/cr9500545

[4] T. Spatzal , M. Aksoyoglu , L. Zhang , S. L. Andrade , E. Schleicher , S. Weber , D. C. Rees , O. Einsle , Science 2011, 334, 940. 22096190 10.1126/science.1214025 PMC3268367

[5] D. V. Yandulov , R. R. Schrock , J. Am. Chem. Soc. 2002, 124, 6252–6253. 12033849 10.1021/ja020186x

[6] D. V. Yandulov , R. R. Schrock , Inorg. Chem. 2005, 44, 1103–1117. 15859292 10.1021/ic040095w

[7] D. V. Yandulov , R. R. Schrock , Science 2003, 301, 76–78. 12843387 10.1126/science.1085326

[8] S. B. Schneider , R. Frankovsky , W. Schnick , Inorg. Chem. 2012, 51, 2366–2373. 22235865 10.1021/ic2023677

[9] G. V. Vajenine , G. Auffermann , Y. Prots , W. Schnelle , R. K. Kremer , A. Simon , R. Kniep , Inorg. Chem. 2001, 40, 4866–4870. 11531432 10.1021/ic010263+

[10] J. C. Crowhurst , A. F. Goncharov , B. Sadigh , C. L. Evans , P. G. Morrall , J. L. Ferreira , A. J. Nelson , Science 2006, 311, 1275–1278. 16513980 10.1126/science.1121813

[11] A. F. Young , C. Sanloup , E. Gregoryanz , S. Scandolo , R. J. Hemley , H. K. Mao , Phys. Rev. Lett. 2006, 96, 155501. 16712167 10.1103/PhysRevLett.96.155501

[12] V. S. Bhadram , D. Y. Kim , T. A. Strobel , Chem. Mater. 2016, 28, 1616–1620.

[13] D. Laniel , G. Weck , P. Loubeyre , Inorg. Chem. 2018, 57, 10685–10693. 30137975 10.1021/acs.inorgchem.8b01325

[14] M. Bykov , E. Bykova , G. Aprilis , K. Glazyrin , E. Koemets , I. Chuvashova , I. Kupenko , C. McCammon , M. Mezouar , V. Prakapenka , H. P. Liermann , F. Tasnadi , A. V. Ponomareva , I. A. Abrikosov , N. Dubrovinskaia , L. Dubrovinsky , Nat. Commun. 2018, 9, 2756. 30013071 10.1038/s41467-018-05143-2 PMC6048061

[15] D. Laniel , A. Dewaele , G. Garbarino , Inorg. Chem. 2018, 57, 6245–6251. 29505253 10.1021/acs.inorgchem.7b03272

[16] D. Laniel , B. Winkler , T. Fedotenko , A. Aslandukova , A. Aslandukov , S. Vogel , T. Meier , M. Bykov , S. Chariton , K. Glazyrin , V. Milman , V. Prakapenka , W. Schnick , L. Dubrovinsky , N. Dubrovinskaia , Phys. Rev. Mater. 2022, 6, 023402.

[17] M. Bykov , K. R. Tasca , I. G. Batyrev , D. Smith , K. Glazyrin , S. Chariton , M. Mahmood , A. F. Goncharov , Inorg. Chem. 2020, 59, 14819–14826. 33000943 10.1021/acs.inorgchem.0c01863

[18] H. Alkhaldi , P. Kroll , J. Phys. Chem. C 2019, 123, 7054–7060.

[19] M. Bykov , T. Fedotenko , S. Chariton , D. Laniel , K. Glazyrin , M. Hanfland , J. S. Smith , V. B. Prakapenka , M. F. Mahmood , A. F. Goncharov , A. V. Ponomareva , F. Tasnadi , A. I. Abrikosov , T. Bin Masood , I. Hotz , A. N. Rudenko , M. I. Katsnelson , N. Dubrovinskaia , L. Dubrovinsky , I. A. Abrikosov , Phys. Rev. Lett. 2021, 126, 175501. 33988447 10.1103/PhysRevLett.126.175501

[20] S. B. Schneider , M. Seibald , V. L. Deringer , R. P. Stoffel , R. Frankovsky , G. M. Friederichs , H. Laqua , V. Duppel , G. Jeschke , R. Dronskowski , W. Schnick , J. Am. Chem. Soc. 2013, 135, 16668–16679. 24090235 10.1021/ja408816t

[21] G. Krach , D. Werhahn , K. Witthaut , D. Johrendt , W. Schnick , Angew. Chem. Int. Ed. 2025, 64, e202420583. 10.1002/anie.202420583 PMC11795704 39512078

[22] C. L. Rom , R. W. Smaha , C. A. Knebel , K. N. Heinselman , J. R. Neilson , S. R. Bauers , A. Zakutayev , J. Mater. Chem. C 2023, 11, 11451–11459.

[23] A. Neuhaus , Chimia 1964, 18, 93–103.

[24] G. Auffermann , Y. Prots , R. Kniep , Angew. Chem. Int. Ed. 2001, 40, 547–549. 10.1002/1521-3773(20010202)40:3<547::AID-ANIE547>3.0.CO;2-X 29712033

[25] M. Bykov , S. Chariton , H. Fei , T. Fedotenko , G. Aprilis , A. V. Ponomareva , F. Tasnadi , I. A. Abrikosov , B. Merle , P. Feldner , S. Vogel , W. Schnick , V. B. Prakapenka , E. Greenberg , M. Hanfland , A. Pakhomova , H. P. Liermann , T. Katsura , N. Dubrovinskaia , L. Dubrovinsky , Nat. Commun. 2019, 10, 2994. 31278267 10.1038/s41467-019-10995-3 PMC6611777

[26] T. Sasaki , T. Yamamoto , S. Asano , K. Niwa , M. Hasegawa , Dalton Trans. 2023, 52, 469–475. 36533452 10.1039/d2dt03433f

[27] Deposition Number(s) 2418207 (for W_2_Be_4_N_5_ at ambient pressure), 2418208 (for BeW_10_N_14_(N_2_) at 17 GPa) contain the supplementary crystallographic data for this paper. These data are provided free of charge by the joint Cambridge Crystallographic Data Centre and Fachinformationszentrum Karlsruhe Access Structures service.

[28] R. J. Angel , Rev. Mineral. Geochem. 2000, 41, 35–59.

[29] A. K. Liang , I. Osmond , G. Krach , L. T. Shi , L. Brüning , U. Ranieri , J. Spender , F. Tasnadi , B. Massani , C. R. Stevens , R. S. McWilliams , E. L. Bright , N. Giordano , S. Gallego‐Parra , Y. Q. Yin , A. Aslandukov , F. I. Akbar , E. Gregoryanz , A. Huxley , M. Peña‐Alvarez , J. G. Si , W. Schnick , M. Bykov , F. Trybel , D. Laniel , Adv. Funct. Mater. 2024, 34, 2313819.

[30] A. Pakhomova , G. Aprilis , M. Bykov , L. Gorelova , S. S. Krivovichev , M. P. Belov , I. A. Abrikosov , L. Dubrovinsky , Nat. Commun. 2019, 10, 2800. 31243286 10.1038/s41467-019-10589-z PMC6594954

[31] D. Spahr , L. Bayarjargal , E. Bykova , M. Bykov , L. Brüning , V. Kovalev , V. Milman , J. Wright , B. Winkler , Inorg. Chem. 2024, 63, 19513–19517. 39383049 10.1021/acs.inorgchem.4c03681

[32] M. M. Pointner , C. Ceniza , L. Nusser , K. Witthaut , F. Wolf , M. Weidemann , L. Eisenburger , A. Moewes , O. Oeckler , W. Schnick , Angew. Chem. Int. Ed. 2024, 63, e202411441. 10.1002/anie.202411441 39041462

[33] K. Niwa , D. Dzivenko , K. Suzuki , R. Riedel , I. Troyan , M. Eremets , M. Hasegawa , Inorg. Chem. 2014, 53, 697–699. 24393052 10.1021/ic402885k

[34] M. Bykov , K. V. Yusenko , E. Bykova , A. Pakhomova , W. Kraus , N. Dubrovinskaia , L. Dubrovinsky , Eur. J. Inorg. Chem. 2019, 2019, 3667–3671.

[35] F. Liebau , Structural Chemistry of Silicates, Springer, Berlin 1985.

[36] N. Casati , A. Genoni , B. Meyer , A. Krawczuk , P. Macchi , Acta Crystallogr. Sect. B 2017, 73, 584–597. 10.1107/S2052520617008356 28762969

[37] D. Tchon , A. Makal , IUCrJ 2021, 8, 1006–1017. 10.1107/S2052252521009532 PMC8562673 34804552

[38] B. Winkler , V. Milman , Z. Kristallogr. Cryst. Mater. 2014, 229, 112–122.

[39] B. Winkler , M. Hytha , M. C. Warren , V. Milman , J. D. Gale , J. Schreuer , Z. Kristallogr. Cryst. Mater. 2001, 216, 67–70.

[40] R. Gaillac , P. Pullumbi , F. X. Coudert , J. Phys. Condens. Matter 2016, 28, 275201. 27199239 10.1088/0953-8984/28/27/275201

[41] M. T. Yeung , R. Mohammadi , R. B. Kaner , Annu. Rev. Mater. Res. 2016, 46, 465–485.

[42] E. Mazhnik , A. R. Oganov , J. Appl. Phys. 2019, 126, 125109.

[43] OriginLab Corporation, OriginPro 2019B, Northhampton (USA), **1991−2019**.

[44] Rigaku Oxford Diffraction , CrysAlisPro Software system, version 171.43.67a, Rigaku Corporation, Oxford (UK) 2023.

[45] A. Aslandukov , M. Aslandukov , N. Dubrovinskaia , L. Dubrovinsky , J. Appl. Crystallogr. 2022, 55, 1383–1391. 36249501 10.1107/S1600576722008081 PMC9533752

[46] F. Birch , Phys. Rev. B 1947, 71, 809–824.

[47] M. R. Buchner , M. Müller , ACS Chem. Health Saf. 2023, 30, 36–43.

[48] Y. Fei , A. Ricolleau , M. Frank , K. Mibe , G. Shen , V. Prakapenka , Proc. Natl. Acad. Sci. USA 2007, 104, 9182–9186. 17483460 10.1073/pnas.0609013104 PMC1890468

[49] J. Gonzalez‐Platas , M. Alvaro , F. Nestola , R. Angel , J. Appl. Crystallogr. 2016, 49, 1377–1382.

[50] H. Huppertz , Z. Kristallogr. Cryst. Mater. 2004, 219, 330–338.

[51] I. Kantor , V. Prakapenka , A. Kantor , P. Dera , A. Kurnosov , S. Sinogeikin , N. Dubrovinskaia , L. Dubrovinsky , Rev. Sci. Instrum. 2012, 83, 125102. 23278021 10.1063/1.4768541

[52] H. P. Liermann , Z. Konôpková , W. Morgenroth , K. Glazyrin , J. Bednarčik , E. E. McBride , S. Petitgirard , J. T. Delitz , M. Wendt , Y. Bican , A. Ehnes , I. Schwark , A. Rothkirch , M. Tischer , J. Heuer , H. Schulte‐Schrepping , T. Kracht , H. Franz , J. Synchrotron Radiat. 2015, 22, 908–924. 26134794 10.1107/S1600577515005937 PMC4489534

[53] K. Momma , F. Izumi , J. Appl. Crystallogr. 2011, 44, 1272–1276.

[54] F. D. Murnaghan , Proc. Natl. Acad. Sci. USA 1944, 30, 244–247. 16588651 10.1073/pnas.30.9.244 PMC1078704

[55] D. Naglav , M. R. Buchner , G. Bendt , F. Kraus , S. Schulz , Angew. Chem. Int. Ed. 2016, 55, 10562–10576. 10.1002/anie.201601809 27364901

[56] G. S. Pawley , J. Appl. Crystallogr. 1981, 14, 357–361.

[57] C. Prescher , V. B. Prakapenka , High Press. Res. 2015, 35, 223–230.

[58] G. M. Sheldrick , Acta Crystallogr. Sect. A Found. Crystallogr. 2015, 71, 3–8. 10.1107/S2053273314026370 PMC4283466 25537383

[59] G. M. Sheldrick , Acta Crystallogr. Sec. C 2015, 71, 3–8. 10.1107/S2053273314026370 PMC4283466 25537383

[60] D. Walker , Am. Mineral. 1991, 76, 1092–1100.

[61] Z. Konopkova , W. Morgenroth , R. Husband , N. Giordano , A. Pakhomova , O. Gutowski , M. Wendt , K. Glazyrin , A. Ehnes , J. T. Delitz , A. F. Goncharov , V. B. Prakapenka , H. P. Liermann , J. Synchrotron Rad. 2021, 28, 1747–1757. 10.1107/S1600577521009231 PMC8570206 34738928

