# Supplementary material for: High‐Pressure Synthesis of Ultra‐Incompressible Beryllium Tungsten Nitride Pernitride BeW10N14(N2)
Source: Angew Chem Int Ed Engl. 2025 Apr 10;64(24):e202505778. doi: 10.1002/anie.202505778 (PMC12144890; doi:10.1002/anie.202505778)
Supplement: Supplementary file 1 — Supporting Information [file ANIE-64-e202505778-s003.pdf]

SUPPORTING INFORMATION

High-Pressure Synthesis of Ultra-Incompressible Beryllium Tungsten Nitride Pernitride $BeW_{10}N_{14}(N_2)$

Georg Krach,[a] Lukas Brüning,[b] Sebastian Ambach,[a] Elena Bykova,[c] Nico Giordano,[d] Björn Winkler,[c] Maxim Bykov,*[b] and Wolfgang Schnick*[a]

[a] G. Krach, Dr. S. J. Ambach, Prof. Dr. W. Schnick
Department of Chemistry, University of Munich (LMU)
Butenandtstraße 5–13, 81377 Munich (Germany)
E-mail: wolfgang.schnick@uni-muenchen.de

[b] L. Brüning, Prof. Dr. M. Bykov
Institute of Inorganic and Analytical Chemistry, Goethe University Frankfurt
Max-von-Laue-Straße 7, 60438 Frankfurt am Main (Germany)
E-Mail: maxim.bykov@chemie.uni-frankfurt.de

[c] Dr. E. Bykova, Prof. Dr. B. Winkler
Institute of Geosciences, Goethe-University Frankfurt
Altenhoeferallee 1, 60438 Frankfurt am Main (Germany)

[d] Dr. N. Giordano
Deutsches Elektronen-Synchrotron DESY
Notkestr. 85, 22607 Hamburg, Germany

Table of Contents

Experimental Procedures **3**
Safety notice 3
High-pressure high-temperature synthesis of $W_2Be_4N_5$ 3
Laser-heated diamond anvil cell (LHDAC) 3
Single crystal X-ray diffraction with Synchrotron radiation 3
Equation of State 4
DFT calculations **4**
Results and Discussion **5**
Redetermination of the crystal structure of $W_2Be_4N_5$ 5
Charge distribution (CHARDI) 7
Elastic properties of $W_2Be_4N_5$ 7
Crystallographic information for $BeW_{10}N_{14}(N_2)$ 8
DFT calculations 13
References **16**

SUPPORTING INFORMATION

Experimental Procedures

Safety notice

Beryllium and its compounds can cause diseases like acute berylliosis, chronic beryllium disease (CBD), contact dermatitis and cancer. Especially the inhalation of Be containing dust or contact with solvable Be salts is dangerous. Consequently, the complete handling of starting materials and Be containing samples was carried out in a designated glovebox. Samples outside the glovebox were only transported in closed vessels. For unavoidable work with Be outside the glovebox (e.g. cleaning working equipment) care must be taken to additional safety precautions like FFP3-masks and the avoidance of dusts. This is especially important for the synthesis of bulk materials used as starting material for the high-pressure experiments. The danger in the Diamond Anvil Cell experiment itself is far lower, since the single crystal we prepared in the experiment exhibits a mass of approx. 5 ng. Guidelines how to work with Be are available in the literature.[1-2]

High-pressure high-temperature synthesis of $W_2Be_4N_5$

The sample of $W_2Be_4N_5$ used for the DAC experiment was synthesized as already described previously in a high-temperature high-pressure approach in a hydraulic 1000 t press utilizing the multianvil technique with a modified Walker module.[3-5] First, a MgO octahedron was prepared as follows: A ZrO_2 cylinder, equipped with two graphite furnaces and MgO spacers was placed in the center of a cylindrical hole drilled into the MgO octahedron (5% Cr_2O_3 doped MgO, 18 mm edge length, Ceramic Substrates & Components Ltd, Isle of Wight). The graphite furnaces were contacted with Mo plates. Stoichiometric amounts of Be_3N_2 (17.0 mg, 0.310 mmol), W (85.4 mg, 0.464 mmol) and NaN_3 (15.1 mg, 0.232 mmol) were ground in a tungsten carbide mortar in a glovebox (see above). The mixture was packed in an *h*-BN crucible (Henze, Kempten) and transferred into the MgO octahedron. The filled octahedron was placed in the void formed by eight tungsten carbide cubes with truncated corners, which were separated with pyrophyllite gaskets. The assembly was transferred to the multianvil apparatus and compressed to 8 GPa within 240 min. At this pressure the sample was heated to 1350 °C within 35 min, kept at this temperature for 15 min and cooled down to room temperature within 15 min before the pressure was decreased to ambient pressure within 720 min. The recovered sample was washed with water to remove residual reactants and analyzed using powder X-ray diffraction. Suitable single crystals were selected and assessed by single-crystal X-ray diffraction.

Laser-heated diamond anvil cell (LHDAC)

High-pressure experiments were carried out in a Boehler-Almax BX90 diamond anvil cell (DAC) with an opening angle of 60°, equipped with a pair of Boehler-Almax diamonds with a culet size of 250 µm.[6] The anvils were separated by a Re gasket with a laser-drilled hole of 100 µm, forming the sample chamber. The cell was loaded with a single crystal of $W_2Be_4N_5$ and a piece of ruby. Ne served as a pressure transmitting medium and pressure gauge. The cell was compressed to 41.0(1) GPa in seven steps at ambient temperature. At each pressure point, sc-XRD data was collected (see below). At the target pressure, the sample was heated from both sides using a near-infrared (NIR) fiber laser (λ = 1064 nm, focused to 20x20 µm^2). After a bright flash occurred, the sample was cooled down to ambient temperature and XRD data were collected. The temperature was likely above 3000 K based on previous observations but was not precisely determined.

Single crystal X-ray diffraction with Synchrotron radiation

Pressure dependent single-crystal X-ray diffraction (sc-XRD) data of $W_2Be_4N_5$ and $BeW_{10}N_{14}(N_2)$ were collected at the Extreme Conditions Beamline P02.2 (PETRA III) at DESY in Hamburg (λ = 0.2905 Å, Perkin Elmer XRD1621 flat panel detector).[7-8] The beam diameter was 1.8x1.4 µm^2. The laser heated sample area was surveyed by acquiring still-images on a regularly spaced grid. Afterwards, on selected points of the grid, sc-XRD data were collected while rotating the DAC around the vertical axis of the goniometer ω in a range from –32° to +32° with a scanning step of 0.5°. For the pressure determination, powder XRD data was obtained by radial integration of single frames using Dioptas.[9] After identification of the reflections of Ne and a subsequent Pawley fit, the pressure was determined using the equation of state of Ne.[10-11]

Data analysis of the multi domain measurements was conducted using the CrysAlisPro software and the Domain Auto Finder.[12-13] Structure solution and subsequent refinement was carried out with SHELXT or SHELXL, respectively.[14-15] Structures were visualized using VESTA.[16]

Equation of State

Using the pressure-volume data from pressure-dependent single crystal refinements of $W_2Be_4N_5$ from ambient pressure to 41.0(1) GPa, the decrease of the unit cell volume was fitted by a 2nd order Birch-Murnaghan equation of state with the program package EoSFit7.[17-19] The results of the DFT calculations for the elastic properties of $BeW_{10}N_{14}(N_2)$ were fitted using a 3rd order Birch-Murnaghan equation of state (Equation 1). The results were plotted using Origin.[20]

$$p(V)=\frac{3}{2}\,K_0\left[\left(\frac{V_0}{V}\right)^{\frac{7}{3}}-\left(\frac{V_0}{V}\right)^{\frac{5}{3}}\right]\cdot\left[1+\frac{3}{4}(K_0'-4)\left\{\left(\frac{V_0}{V}\right)^{-\frac{2}{3}}-1\right\}\right] \qquad (1)$$

(K_0: isothermal bulk modulus, V_0: unit cell volume at a theoretical pressure of 0 GPa, V: unit cell volume, K_0' partial pressure derivative of the isothermal bulk modulus)

DFT calculations

First-principles calculations were carried out within the framework of density functional theory (DFT), employing the Perdew−Burke−Ernzerhof (PBE) exchange-correlation functional and the plane wave/pseudopotential approach implemented in the CASTEP simulation package.[21-23] “On the fly” norm-conserving pseudopotentials generated using the descriptors in the CASTEP database, were employed in conjunction with plane waves up to a kinetic energy cutoff of 990 eV. The accuracy of the pseudopotentials is well established.[24] A Monkhorst−Pack grid was used for Brillouin zone integrations.[25] We used a distance between grid points of <0.023 $Å^{-1}$. Convergence criteria for geometry optimization included an energy change of <5 × 10−6 eV $atom^{-1}$ between steps, a maximal force of <0.01 eV $Å^{-1}$ and a maximal component of the stress tensor <0.02 GPa. The elastic stiffness tensor was computed by stress-strain calculations.

Results and Discussion

Redetermination of the crystal structure of $W_2Be_4N_5$

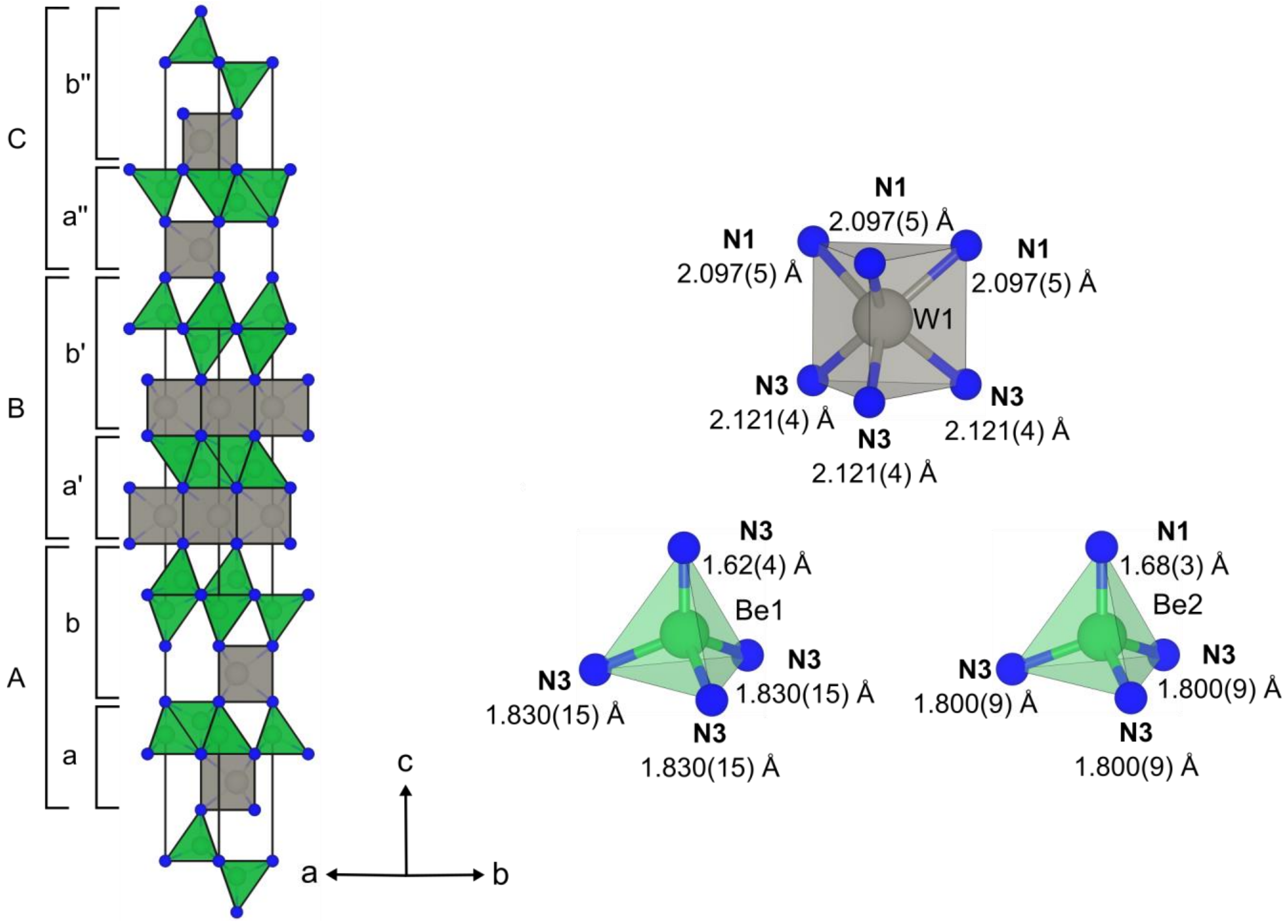

Figure S1. Refined crystal structure of $W_2Be_4N_5$ along [001] and the coordination polyhedra W1, Be1 and Be2 (left). The structure consists of layers of WN_6 trigonal prisms that are separated by layers of edge-sharing BeN_4 tetrahedra. W: gray; Be: green, N: blue.

The redetermination of the crystal structure of $W_2Be_4N_5$ at ambient pressure confirmed the structural model, which we had found before. A detailed description of the crystal structure was already reported elsewhere.[3] However, it is worth mentioning, that the bonding distance Be1–N3 of 1.62(4) Å is longer than reported before (1.53(3) Å) and identical to the bond distance Be2–N1 of 1.68(3) Å within the experimental errors. These findings are consistent with the more precise CHARDI calculations of $W_2Be_4N_5$, which can be found in Table S4.

Table S1. Crystallographic data of $W_2Be_4N_5$. Standard deviations are given in parentheses.

**Formula**	$\mathbf{W_2Be_4N_5}$
Crystal system	trigonal
Space group	$R\bar{3}m$ (no. 166)
Lattice parameters / Å	a = 2.8702(2)
	c = 37.1324(18)
Cell volume / $Å^3$	264.92(2)
Formula units per unit cell	3
Density / g cm^{-3}	8.909
Molecular weight / g mol^{-1}	473.79
Linear absorption coefficient / mm^{-1}	6.197
Diffractometer	P02.2 (DESY)
Wavelength / Å	0.2905
Absorption correction	multi-scan
F(000)	597
θ range / °	$4.032 \le \theta \le 17.537$
Total no. of reflections	318
Independent reflections [$I \ge 2\sigma(I)$/all]	175/181
R_{int}	0.017
Refined parameters	17
Goodness of fit	1.085
R-values [$I \ge 2\sigma(I)$]	R_1 = 0.0254; $wR2$ = 0.0617
R-values [all data]	R_1 = 0.0269; $wR2$ = 0.0625
$\Delta\rho_{max}$, $\Delta\rho_{min}$ / e $Å^3$	4.28; –2.92

Table S2. Wyckoff positions, coordinates, isotropic thermal displacement parameters and occupancies in $W_2Be_4N_5$. Standard deviations are given in parentheses.

**Atom**	**Wyck.**	x	y	z	$\mathbf{U_{eq}}$ **/ $Å^2$**	**Occ.**
W1	6*c*	0	0	0.06791(2)	0.0056(1)	1
N1	6*c*	0	0	0.2308(2)	0.0072(10)	1
N2	3*c*	0	0	1/2	0.0074(14)	1
N3	6*c*	0	0	0.30107(17)	0.0048(9)	1
Be1	6*c*	0	0	0.3447(9)	0.046(7)	1
Be2	6*c*	0	0	0.1856(6)	0.028(4)	1

Table S3. Anisotropic displacement parameters for W and N in $W_2Be_4N_5$. Standard deviations are given in parentheses.

Atom	U_{11} / Å^2	U_{22} / Å^2	U_{33} / Å^2	U_{12} / Å^2	U_{13} / Å^2	U_{23} / Å^2
W1	0.00245(16)	0.00245(16)	0.01176(19)	0.00123(8)	0	0
N1	0.0048(17)	0.0048(17)	0.012(2)	0.0024(9)	0	0
N2	0.006(2)	0.006(2)	0.010(3)	0.0029(12)	0	0
N3	0.0041(15)	0.0041(15)	0.0062(19)	0.0021(8)	0	0

Charge distribution (CHARDI)

Table S4: Results of the CHARDI analysis for $W_2Be_4N_5$.

Polyhedron	W N1 N1 N1 N3 N3 N3	Be1 N3 N3 N3 N3	Be2 N1 N2 N2 N2
Average bong length / Å	2.1091	1.774	1.7697
Polyhedral volume / Å^3	9.3078	2.8490	2.8317
Distortion index (bond length)	0.00557	0.04424	0.02579
Quadratic elongation	-	1.0102	1.0038
Bond angle variance / °^{2}	-	41.7124	15.8005
Effective coordination number	5.9936	3.3456	3.8277
**Total charge (theory)**			
W / Be	3.570 (3.500)	1.741 (2.000)	2.305 (2.000)
N1	-2.472(-3)		-2.472
N2	-3.691 (-3)		-2.674
N3		-3.4095 (-3)	

Elastic properties of $W_2Be_4N_5$

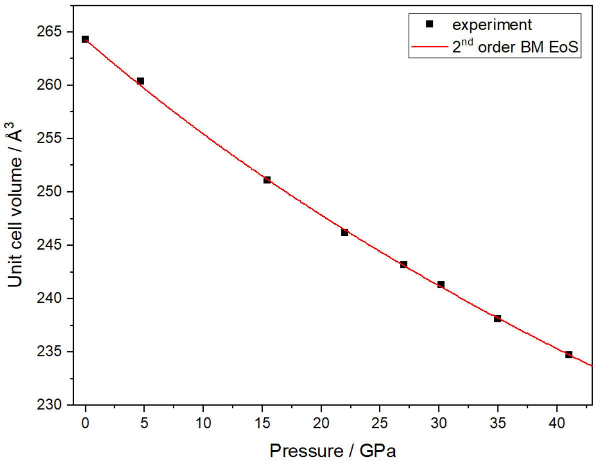

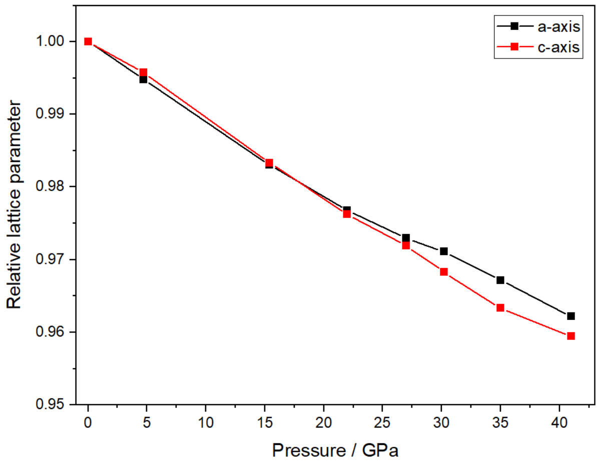

Figure S2. Pressure-volume data with a fit using a 2nd order Birch-Murnaghan equation of state (left) and the evolution of the lattice parameters of $W_2Be_4N_5$ with increasing pressure (right). Error bars are smaller than the symbol size.

The bulk modulus of $W_2Be_4N_5$ was determined using the pressure-volume data based on sc-XRD refinements at different pressure points. The data was fitted with a 2nd order Birch-Murnaghan equation of state (Figure S2) with the fitting parameters V_0 = 264.28(2) Å^3, K_0 = 273.4(11) GPa and a fixed value for K' of 4.0. Additionally, the relative evolution of the lattice parameters with increasing pressure was examined (Figure S2). The pressure-induced shortening of the lattice parameters is similar for the *a*- and *c*-axis until about 25 GPa, when the *c*-axis becomes slightly more compressible. No pressure-induced phase transition was observed and $W_2Be_4N_5$ stays stable up to 41.0(1) GPa on cold compression. At this point, the sample was heated.

Crystallographic information for $BeW_{10}N_{14}(N_2)$

Table S5. Crystallographic data of $BeW_{10}N_{14}(N_2)$ at different pressure points. Standard deviations are given in parentheses.

**Formula**		**$BeW_{10}N_{14}(N_2)$**	
Crystal system		monoclinic	
Space group		*C*2/*m* (no. 12)	
Lattice parameters / Å, °	*a* = 20.69(3)	*a* = 20.554(2)	*a* = 19.803(5)
	b = 2.8666(4)	*b* = 2.8457(4)	*b* = 2.7867(6)
	c = 8.539(12)	*c* = 8.324(7)	*c* = 8.2467(15)
	β = 100.69(15)	*β* = 99.59(3)	*β* = 99.605(19)
Cell volume /Å^3	497.7(1)	480.1(4)	448.72(17)
Pressure / GPa	0	16.5(1)	42.1(1)
Formula units per unit cell		2	
Density / g cm^{-3}	13.824	14.331	15.332
Molecular weight / g mol^{-1}		2071.67	
Linear absorption coefficient / mm^{-1}	10.958	11.359	12.154
Beamline		P02.2 (DESY)	
Wavelength / Å		0.2905	
F(000)		1712	
θ range / °	2.327 ≤ *θ* ≤ 17.291	1.643 ≤ *θ* ≤ 17.797	2.436 ≤ *θ* ≤ 17.728
Total no. of reflections	758	1006	926
Independent reflections [$I \geq 2\sigma(I)$/all]	256/510	397/577	478/591
R_{int}	0.090	0.043	0.057
Refined parameters	49	56	56
Goodness of fit	1.053	0.968	1.137
R-values [$I \geq 2\sigma(I)$]	R_1 = 0.087; *wR*2 = 0.157	R_1 = 0.046; *wR*2 = 0.097	R_1 = 0.064; *wR*2 = 0.148
R-values [all data]	R_1 = 0.189; *wR*2 = 0.197	R_1 = 0.083; *wR*2 = 0.109	R_1 = 0.082; *wR*2 = 0.164
$\Delta\rho_{max}$, $\Delta\rho_{min}$ / e Å^3	5.07; –5.21	3.93; –3.42	6.15; –4.71

Table S6. Wyckoff positions, coordinates, isotropic thermal displacement parameters and occupancies in $BeW_{10}N_{14}(N_2)$ at 16.5(1) GPa. Standard deviations are given in parentheses.

**Atom**	**Wyck.**	***x***	***y***	***z***	**U_{eq} / Å^2**	**Occ.**
W1	4*i*	0.17043(5)	0	0.4744(3)	0.0057(7)	1
W2	4*i*	0.55455(5)	0	0.3115(3)	0.0064(7)	1
W3	4*i*	0.62640(5)	0	0.0433(3)	0.0064(7)	1
W4	4*i*	0.09242(5)	0	0.7337(3)	0.0073(7)	1
W5	4*i*	0.76901(5)	0	0.1974(3)	0.0130(10)	1
N1	4*i*	0.2622(9)	0	0.381(6)	0.006(3)	1
N2	4*i*	0.6544(10)	0	0.310(7)	0.012(4)	1
N3	4*i*	0.0620(11)	0	0.121(8)	0.012(4)	1
N4	4*i*	0.0000(12)	0	0.418(7)	0.013(4)	1
N5	4*i*	0.3951(10)	0	0.458(6)	0.007(3)	1
N6	4*i*	0.3402(9)	0	0.160(6)	0.008(3)	1
N7	4*i*	0.2052(11)	0	0.080(7)	0.013(4)	1
N8	4*i*	0.4563(11)	0	0.167(7)	0.012(4)	1
Be1	2*b*	0	1/2	0	0.05(3)	1

Table S7. Anisotropic displacement parameters for W in $BeW_{10}N_{14}(N_2)$ at 16.5(1) GPa. Standard deviations are given in parentheses.

Atom	U_{11} / Å^2	U_{22} / Å^2	U_{33} / Å^2	U_{12} / Å^2	U_{13} / Å^2	U_{23} / Å^2
W1	0.0103(4)	0.0046(4)	0.002(2)	0	0.0007(6)	0
W2	0.0104(4)	0.0043(4)	0.004(2)	0	-0.0006(6)	0
W3	0.0104(4)	0.0045(4)	0.004(2)	0	0.0006(6)	0
W4	0.0107(4)	0.0053(4)	0.005(2)	0	-0.0009(6)	0
W5	0.0144(4)	0.0084(4)	0.014(3)	0	-0.0036(7)	0

Table S8. Wyckoff positions, coordinates, isotropic thermal displacement parameters and occupancies in $BeW_{10}N_{14}(N_2)$ at 42.1(1) GPa Standard deviations are given in parentheses.

Atom	Wyck.	*x*	*y*	*z*	U_{eq} / Å^2	Occ.
W1	4*i*	0.17015(9)	0	0.4671(2)	0.0078(5)	1
W2	4*i*	0.55232(9)	0	0.3015(2)	0.0079(5)	1
W3	4*i*	0.62261(9)	0	0.0292(2)	0.0074(5)	1
W4	4*i*	0.09578(9)	0	0.7250(2)	0.0077(5)	1
W5	4*i*	0.76122(11)	0	0.2089(3)	0.0147(7)	1
N1	4*i*	0.266(2)	0	0.386(4)	0.008(5)	1
N2	4*i*	0.6582(19)	0	0.293(4)	0.007(5)	1
N3	4*i*	0.0590(18)	0	0.113(4)	0.006(4)	1
N4	4*i*	-0.002(3)	0	0.416(6)	0.017(7)	1
N5	4*i*	0.398(2)	0	0.463(6)	0.015(7)	1
N6	4*i*	0.336(2)	0	0.178(5)	0.012(6)	1
N7	4*i*	0.201(2)	0	0.059(5)	0.012(6)	1
N8	4*i*	0.456(2)	0	0.160(5)	0.011(5)	1
Be1	2*b*	0	1/2	0	0.09(9)	1

Table S9. Anisotropic displacement parameters for W in $BeW_{10}N_{14}(N_2)$ at 42.1(1) GPa. Standard deviations are given in parentheses.

Atom	U_{11} / Å^2	U_{22} / Å^2	U_{33} / Å^2	U_{12} / Å^2	U_{13} / Å^2	U_{23} / Å^2
W1	0.0087(11)	0.0056(9)	0.0091(8)	0	0.0016(7)	0
W2	0.0109(11)	0.0047(9)	0.0080(8)	0	0.0014(7)	0
W3	0.0089(11)	0.0056(9)	0.0076(8)	0	0.0013(7)	0
W4	0.0107(11)	0.0047(9)	0.0075(8)	0	0.0011(7)	0
W5	0.0160(14)	0.0105(11)	0.0166(11)	0	-0.0004(9)	0

Table S10. Wyckoff positions, coordinates, isotropic thermal displacement parameters and occupancies in $BeW_{10}N_{14}(N_2)$ at 0 GPa. Standard deviations are given in parentheses.

Atom	Wyck.	*x*	*y*	*z*	U_{eq} / Å^2	Occ.
W1	4*i*	0.1701(3)	0	0.4712(8)	0.010(2)	1
W2	4*i*	0.5531(3)	0	0.3080(8)	0.012(3)	1
W3	4*i*	0.6232(3)	0	0.0356(8)	0.012(2)	1
W4	4*i*	0.0950(3)	0	0.7268(8)	0.011(2)	1
W5	4*i*	0.7653(6)	0	0.2015(12)	0.034(4)	1
N1	4*i*	0.264(7)	0	0.385(15)	0.006(3)	1
N2	4*i*	0.646(6)	0	0.298(15)	0.006(3)	1
N3	4*i*	0.060(6)	0	0.118(14)	0.006(3)	1
N4	4*i*	-0.006(6)	0	0.402(14)	0.006(3)	1
N5	4*i*	0.379(6)	0	0.411(15)	0.006(3)	1
N6	4*i*	0.334(7)	0	0.158(16)	0.006(3)	1
N7	4*i*	0.184(6)	0	0.034(15)	0.006(3)	1
N8	4*i*	0.457(7)	0	0.178(15)	0.006(3)	1
Be1	2*b*	0	1/2	0	0.11(16)	1

Table S11. Anisotropic displacement parameters for W in $BeW_{10}N_{14}(N_2)$ at 0 GPa. Standard deviations are given in parentheses.

Atom	U_{11} / Å^2	U_{22} / Å^2	U_{33} / Å^2	U_{12} / Å^2	U_{13} / Å^2	U_{23} / Å^2
W1	0.005(4)	0.0005(7)	0.026(5)	0	0.007(5)	0
W2	0.016(5)	0.0025(8)	0.018(6)	0	0.008(5)	0
W3	0.014(5)	0.0036(8)	0.021(5)	0	0.008(5)	0
W4	0.013(5)	0.0029(8)	0.017(5)	0	0.004(5)	0
W5	0.029(9)	0.0055(10)	0.059(8)	0	-0.012(7)	0

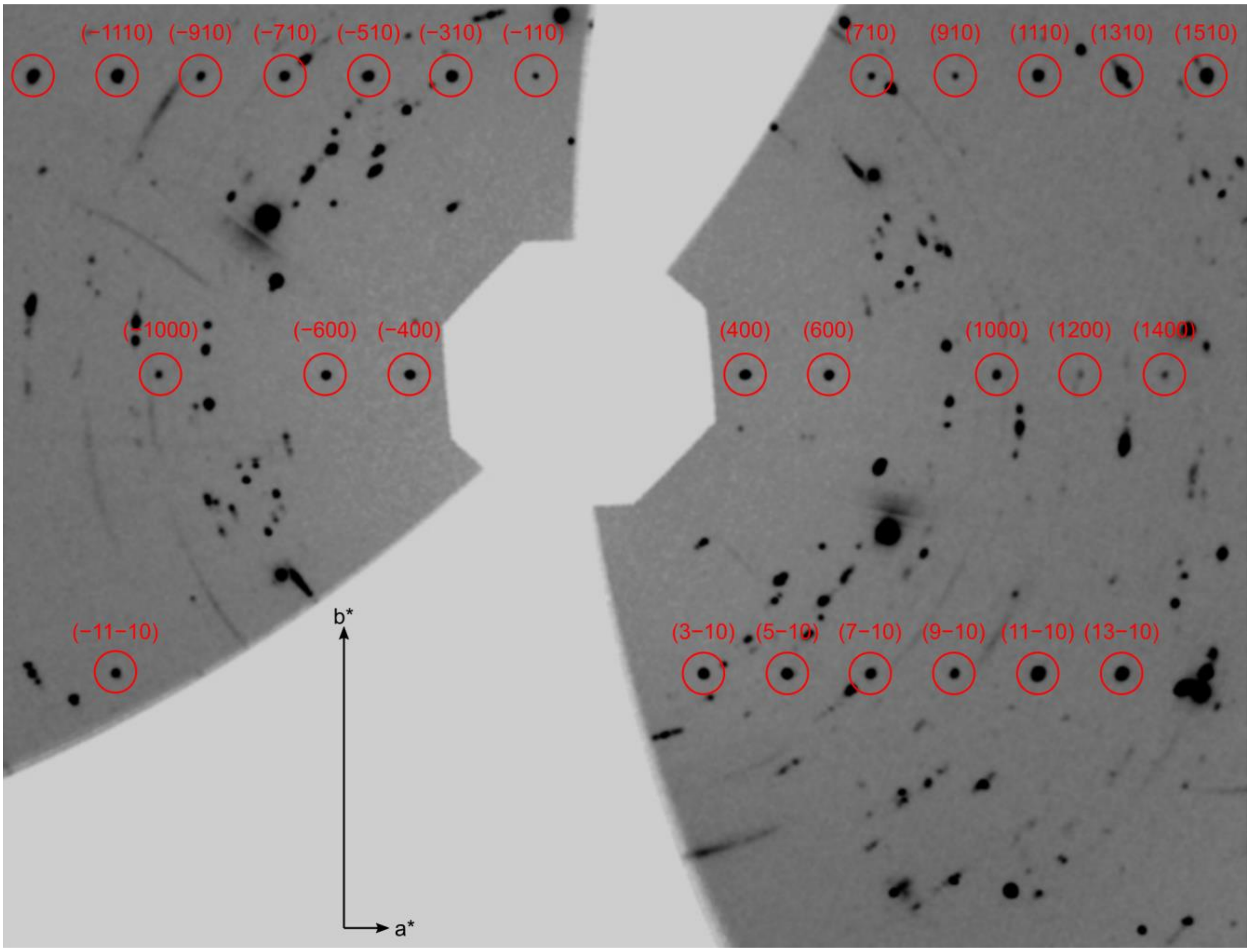

Figure S3. (*hk*0)-reciprocal lattice plane of $BeW_{10}N_{14}(N_2)$ at 16.5(1) GPa with indexed reflections. All reflections fulfill the reflection condition $h + k = 2n$ for the *C*-centering. Additional reflections arise from unreacted starting material $W_2Be_4N_5$, the pressure transmitting medium Ne and the side phase W_2N_3.[3, 26]

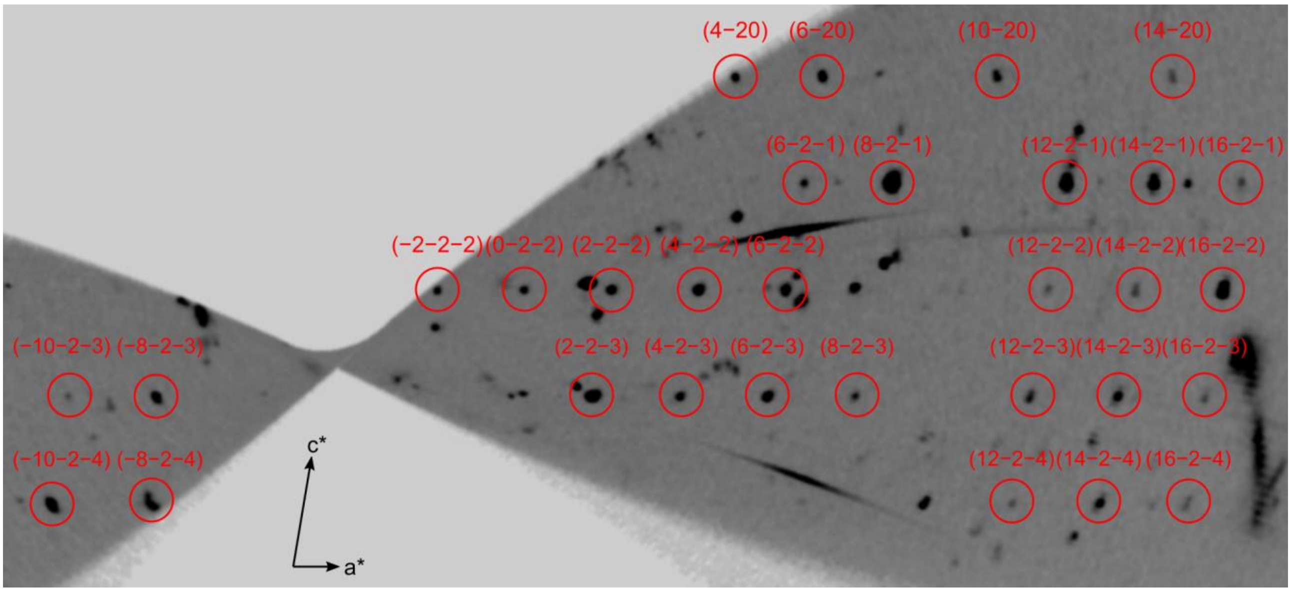

Figure S4. (*h*−2*l*)-reciprocal lattice plane of $BeW_{10}N_{14}(N_2)$ at 16.5(1) GPa with indexed reflections. All reflections fulfill the reflection condition $h + k = 2n$ for the *C*-centering. Additional reflections arise from unreacted starting material $W_2Be_4N_5$, the pressure transmitting medium Ne and the side phase W_2N_3.[3, 26]

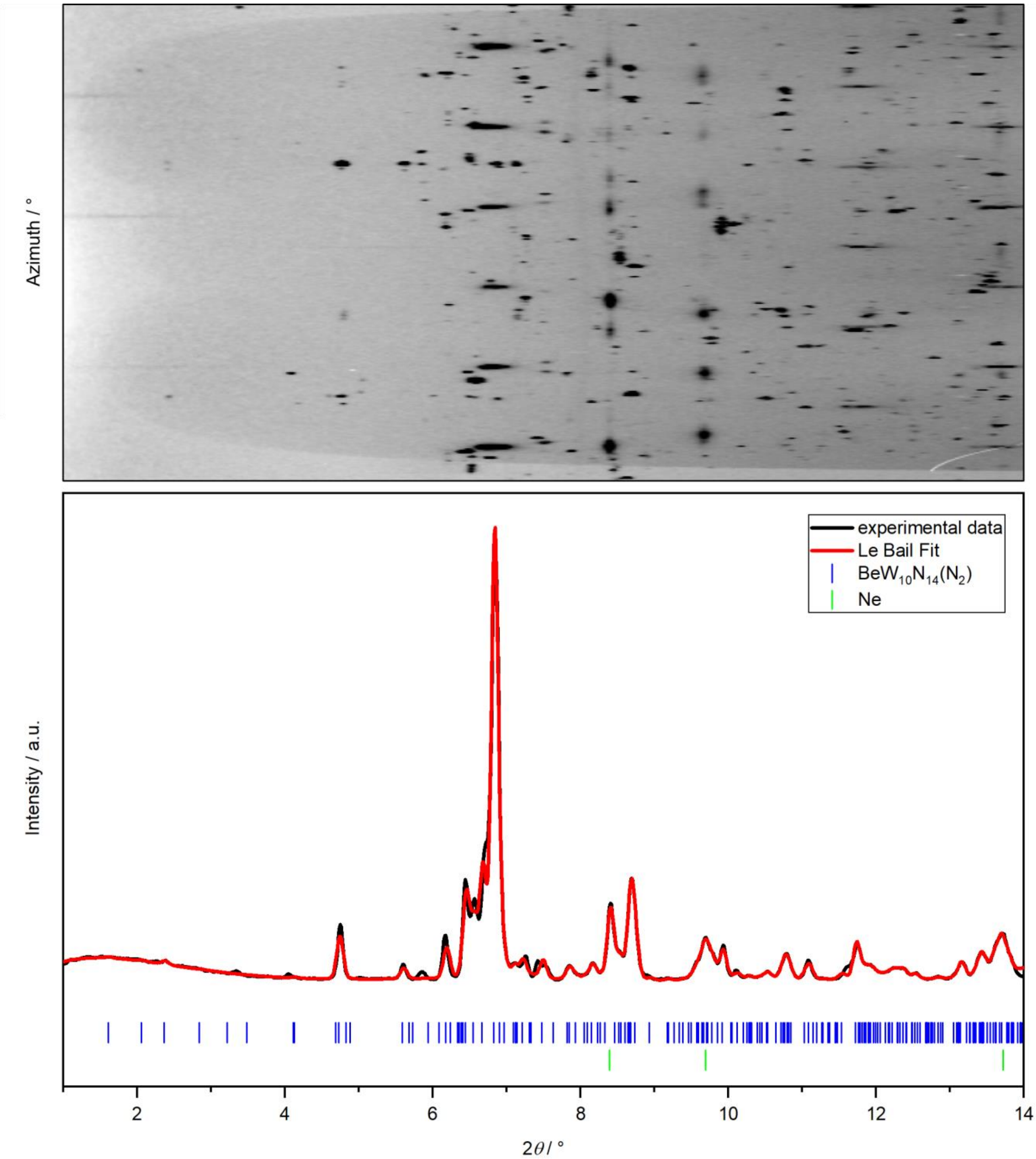

Figure S5. Azimuthal integrated data, powder diffraction pattern and Le Bail fit of $BeW_{10}N_{14}(N_2)$ at 16.5(1) GPa. Positions of Bragg reflections of $BeW_{10}N_{14}(N_2)$ and Ne are given as blue and green bars, respectively (λ = 0.2905 Å).

DFT calculations

$$\begin{pmatrix} 537.29 & 208.7 & 240 & 0 & 21.545 & 0 \\ 208.7 & 585.34 & 289.76 & 0 & -49.144 & 0 \\ 240 & 289.76 & 433.44 & 0 & 25.453 & 0 \\ 0 & 0 & 0 & 184.36 & 0 & -41.033 \\ 21.545 & -49.144 & 25.453 & 0 & 180.86 & 0 \\ 0 & 0 & 0 & -41.033 & 0 & 174.95 \end{pmatrix}$$

Figure S6. Elastic stiffness matrix for $BeW_{10}N_{14}(N_2)$. Values are given in GPa. Its evaluation with the program ELATE reveals an isothermal bulk modulus $K_0 = 337$ GPa, implying that this compound is ultra-incompressible. [27]

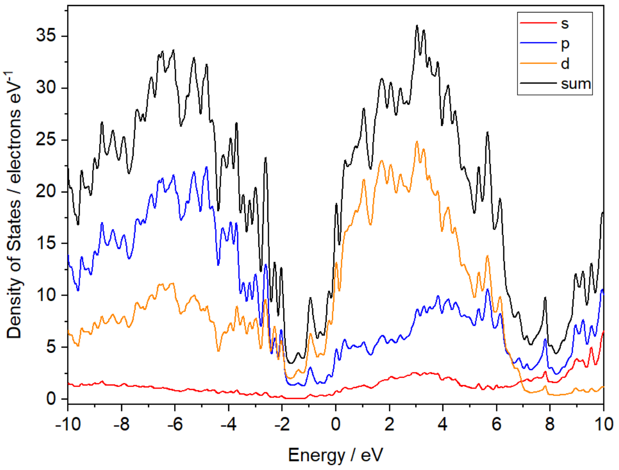

Figure S7. Density of States of $BeW_{10}N_{14}(N_2)$. The calculation shows no band gap and thus, the system is metallic.

Table S12. Calculated Mulliken charges in $BeW_{10}N_{14}(N_2)$. The calculated value of N4 differs from the values of the other nitrogen atoms due to the formation of the (N_2) dumbbell.

Atoms	Mulliken charge
W1	0.914
W2	0.920
W3	0.901
W4	0.937
W5	0.943
N1	-0.610
N2	-0.597
N3	-0.684
N4*	-0.447
N5	-0.613
N6	-0.606
N7	-0.667
N8	-0.665
Be1	0.545

Table S13. Crystallographic data of $BeW_{10}N_{14}(N_2)$ at 0 and 45 GPa derived from DFT calculations.

**Formula**		**$BeW_{10}N_{14}(N_2)$**
Crystal system	monoclinic	
Space group	*C*2/*m* (no. 12)	
Lattice parameters / Å, °	*a* = 21.2258	*a* = 20.4120
	b = 2.8415	*b* = 2.7636
	c = 8.5983	*c* = 8.2749
	β = 100.9854	*β* = 100.7552
Cell volume /Å^3	509.09	458.59
Pressure / GPa	0	45
Formula units per unit cell	2	
Density / g cm^{-3}	13.514	15.002
Molecular weight / g mol^{-1}	2071.67	

Table S14. Wyckoff positions, coordinates, isotropic thermal displacement parameters and occupancies in $BeW_{10}N_{14}(N_2)$ at 0 GPa derived from DFT calculations.

**Atom**	**Wyck.**	***x***	***y***	***z***	**U_{eq} / Å^2**	**Occ.**
W1	4*i*	0.16876	0	0.47309	0.0060	1
W2	4*i*	0.55300	0	0.31178	0.0072	1
W3	4*i*	0.62616	0	0.04524	0.0067	1
W4	4*i*	0.09305	0	0.73310	0.0077	1
W5	4*i*	0.76901	0	0.20407	0.0138	1
N1	4*i*	0.26046	0	0.38369	0.007	1
N2	4*i*	0.64902	0	0.29819	0.013	1
N3	4*i*	0.05896	0	0.11910	0.012	1
N4	4*i*	0.00045	0	0.58416	0.013	1
N5	4*i*	0.39522	0	0.45979	0.008	1
N6	4*i*	0.33833	0	0.16383	0.007	1
N7	4*i*	0.20431	0	0.07935	0.013	1
N8	4*i*	0.45459	0	0.15486	0.011	1
Be1	2*b*	0	1/2	0	0.100	1

Table S15. Anisotropic displacement parameters for W in $BeW_{10}N_{14}(N_2)$ at 0 GPa derived from DFT calculations.

**Atom**	**U_{11} / Å^2**	**U_{22} / Å^2**	**U_{33} / Å^2**	**U_{12} / Å^2**	**U_{13} / Å^2**	**U_{23} / Å^2**
W1	0.01099	0.00449	0.00212	0.00000	0.00053	0.00000
W2	0.01099	0.00439	0.00529	0.00000	-0.00053	0.00000
W3	0.01099	0.00449	0.00423	0.00000	0.00063	0.00000
W4	0.01142	0.00528	0.00529	0.00000	-0.00116	0.00000
W5	0.01543	0.00838	0.01481	0.00000	-0.00402	0.00000

Table S16. Wyckoff positions, coordinates, isotropic thermal displacement parameters and occupancies in $BeW_{10}N_{14}(N_2)$ at 45 GPa derived from DFT calculations.

Atom	Wyck.	*x*	*y*	*z*	U_{eq} / Å^2	Occ.
W1	4*i*	0.16967	0	0.47258	0.0055	1
W2	4*i*	0.55283	0	0.30810	0.0067	1
W3	4*i*	0.62520	0	0.04096	0.0062	1
W4	4*i*	0.09292	0	0.73037	0.0071	1
W5	4*i*	0.76811	0	0.19980	0.0128	1
N1	4*i*	0.26030	0	0.38380	0.007	1
N2	4*i*	0.65183	0	0.29241	0.013	1
N3	4*i*	0.05773	0	0.11495	0.012	1
N4	4*i*	0.00015	0	0.58396	0.013	1
N5	4*i*	0.39614	0	0.46012	0.008	1
N6	4*i*	0.33786	0	0.16763	0.007	1
N7	4*i*	0.20467	0	0.06757	0.013	1
N8	4*i*	0.45621	0	0.15616	0.010	1
Be1	2*b*	0	1/2	0	0.010	1

Table S17. Anisotropic displacement parameters for W in $BeW_{10}N_{14}(N_2)$ at 45 GPa derived from DFT calculations.

Atom	U_{11} / Å^2	U_{22} / Å^2	U_{33} / Å^2	U_{12} / Å^2	U_{13} / Å^2	U_{23} / Å^2
W1	0.01018	0.00424	0.00196	0.00000	0.00049	0.00000
W2	0.01018	0.00415	0.00491	0.00000	-0.00049	0.00000
W3	0.01018	0.00424	0.00392	0.00000	0.00059	0.00000
W4	0.01057	0.00500	0.00491	0.00000	-0.00108	0.00000
W5	0.01429	0.00792	0.01373	0.00000	-0.00372	0.00000

References

[1] M. R. Buchner, M. Müller, *ACS Chem. Health Saf.* **2023**, *30*, 36-43.
[2] D. Naglav, M. R. Buchner, G. Bendt, F. Kraus, S. Schulz, *Angew. Chem. Int. Ed.* **2016**, *55*, 10562-10576.
[3] G. Krach, D. Werhahn, K. Witthaut, D. Johrendt, W. Schnick, *Angew. Chem. Int. Ed.* **2025**, *64*, e202420583.
[4] H. Huppertz, *Z. Kristallogr. Cryst. Mater.* **2004**, *219*, 330-338.
[5] D. Walker, *Am. Mineral.* **1991**, *76*, 1092-1100.
[6] I. Kantor, V. Prakapenka, A. Kantor, P. Dera, A. Kurnosov, S. Sinogeikin, N. Dubrovinskaia, L. Dubrovinsky, *Rev. Sci. Instrum.* **2012**, *83*, 125102.
[7] H. P. Liermann, Z. Konôpková, W. Morgenroth, K. Glazyrin, J. Bednarčik, E. E. McBride, S. Petitgirard, J. T. Delitz, M. Wendt, Y. Bican, A. Ehnes, I. Schwark, A. Rothkirch, M. Tischer, J. Heuer, H. Schulte-Schrepping, T. Kracht, H. Franz, *J. Synchrotron Radiat.* **2015**, *22*, 908-924.
[8] Z. Konopkova, W. Morgenroth, R. Husband, N. Giordano, A. Pakhomova, O. Gutowski, M. Wendt, K. Glazyrin, A. Ehnes, J. T. Delitz, A. F. Goncharov, V. B. Prakapenka, H. P. Liermann, *J. Synchrotron Rad.* **2021**, *28*, 1747-1757.
[9] C. Prescher, V. B. Prakapenka, *High Press. Res.* **2015**, *35*, 223-230.
[10] G. S. Pawley, *J. Appl. Crystallogr.* **1981**, *14*, 357-361.
[11] Y. Fei, A. Ricolleau, M. Frank, K. Mibe, G. Shen, V. Prakapenka, *PNAS* **2007**, *104*, 9182-9186.
[12] Rigaku Oxford Diffraction, *CrysAlisPro Software system, version 171.43.67a*, Rigaku Corporation, Oxford (UK), **2023**.
[13] A. Aslandukov, M. Aslandukov, N. Dubrovinskaia, L. Dubrovinsky, *J. Appl. Crystallogr.* **2022**, *55*, 1383-1391.
[14] G. M. Sheldrick, *Acta Crystallogr. Sect. A* **2015**, *71*, 3-8.
[15] G. M. Sheldrick, *Acta Crystallogr. Sec. C* **2015**, *71*, 3-8.
[16] K. Momma, F. Izumi, *J. Appl. Crystallogr.* **2011**, *44*, 1272-1276.
[17] F. Birch, *Phys. Rev. B* **1947**, *71*, 809-824.
[18] F. D. Murnaghan, *PNAS* **1944**, *30*, 244-247.
[19] J. Gonzalez-Platas, M. Alvaro, F. Nestola, R. Angel, *J. Appl. Crystallogr.* **2016**, *49*, 1377-1382.
[20] OriginLab Corporation, OriginPro 2019B, Northhampton (USA), **1991−2019**.
[21] P. Hohenberg and W. Kohn, *Phys. Rev. B*, **1964**, 136, 864-871.
[22] J. P. Perdew, K. Burke, M. Ernzerhof, *Phys. Rev. Lett.* **1996**, *77*, 3865-3868.
[23] S. J. Clark, M. D. Segall, C. J. Pickard, P. J. Hasnip, M. I. J. Probert, K. Refson, M. C. Payne, *Z. Kristallogr.* **2005**, *220*, 567-570.
[24] K. Lejaeghere, G. Bihlmayer, T. Bjorkman, P. Blaha, S. Blugel, V. Blum, D. Caliste, I. E. Castelli, S. J. Clark, A. Dal Corso, S. de Gironcoli, T. Deutsch, J. K. Dewhurst, I. Di Marco, C. Draxl, M. Dulak, O. Eriksson, J. A. Flores-Livas, K. F. Garrity, L. Genovese, P. Giannozzi, M. Giantomassi, S. Goedecker, X. Gonze, O. Granas, E. K. Gross, A. Gulans, F. Gygi, D. R. Hamann, P. J. Hasnip, N. A. Holzwarth, D. Iusan, D. B. Jochym, F. Jollet, D. Jones, G. Kresse, K. Koepernik, E. Kucukbenli, Y. O. Kvashnin, I. L. Locht, S. Lubeck, M. Marsman, N. Marzari, U. Nitzsche, L. Nordstrom, T. Ozaki, L. Paulatto, C. J. Pickard, W. Poelmans, M. I. Probert, K. Refson, M. Richter, G. M. Rignanese, S. Saha, M. Scheffler, M. Schlipf, K. Schwarz, S. Sharma, F. Tavazza, P. Thunstrom, A. Tkatchenko, M. Torrent, D. Vanderbilt, M. J. van Setten, V. Van Speybroeck, J. M. Wills, J. R. Yates, G. X. Zhang, S. Cottenier, *Science* **2016**, *351*, aad3000.
[25] H. J. Monkhorst, J. D. Pack, *Phys. Rev. B* **1976**, *13*, 5188-5192.
[26] A. K. Liang, I. Osmond, G. Krach, L. T. Shi, L. Brüning, U. Ranieri, J. Spender, F. Tasnadi, B. Massani, C. R. Stevens, R. S. McWilliams, E. L. Bright, N. Giordano, S. Gallego-Parra, Y. Q. Yin, A. Aslandukov, F. I. Akbar, E. Gregoryanz, A. Huxley, M. Peña-Alvarez, J. G. Si, W. Schnick, M. Bykov, F. Trybel, D. Laniel, *Adv. Funct. Mater.* **2024**, *34*, 2313819.
[27] R. Gaillac, P. Pullumbi, F. X. Coudert, *J. Phys. Condens. Matter* **2016**, *28*, 275201.
